# Evaluation of a national lung cancer symptom awareness campaign in Wales

**DOI:** 10.1038/s41416-019-0676-2

**Published:** 2019-12-16

**Authors:** Grace McCutchan, Stephanie Smits, Lucy Ironmonger, Ciarán Slyne, Amanda Boughey, Jodie Moffat, Rebecca Thomas, Dyfed Wyn Huws, Kate Brain

**Affiliations:** 1 Cardiff University, School of Medicine, Division of Population Medicine, Cardiff, UK; 2 Cancer Research UK, London, UK; 3 Cwm Taf Morgannwg University Health Board, Keir Hardie University Health Park,, Merthyr Tydfil, UK; 4 Formerly Cancer Research UK, London, UK; 5 Public Health Wales, Welsh Cancer Intelligence and Surveillance Unit, Cardiff, UK

**Keywords:** Respiratory signs and symptoms, Human behaviour, Public health, Signs and symptoms

## Abstract

**Background:**

Lung cancer is the leading cause of cancer mortality in Wales. We conducted a before- and after- study to evaluate the impact of a four-week mass-media campaign on awareness, presentation behaviour and lung cancer outcomes.

**Methods:**

Population-representative samples were surveyed for cough symptom recall/recognition and worry about wasting doctors’ time pre-campaign (June 2016; *n* = 1001) and post-campaign (September 2016; *n* = 1013). GP cough symptom visits, urgent suspected cancer (USC) referrals, GP-ordered radiology, new lung cancer diagnoses and stage at diagnosis were compared using routine data during the campaign (July–August 2016) and corresponding control (July–August 2015) periods.

**Results:**

Increased cough symptom recall (*p* < 0.001), recognition (*p* < 0.001) and decreased worry (*p* < 0.001) were observed. GP visits for cough increased by 29% in the target 50+ age-group during the campaign (*p* < 0.001) and GP-ordered chest X-rays increased by 23% (*p* < 0.001). There was no statistically significant change in USC referrals (*p* = 0.82), new (*p* = 0.70) or early stage (*p* = 0.27) diagnoses, or in routes to diagnosis.

**Conclusions:**

Symptom awareness, presentation and GP-ordered chest X-rays increased during the campaign but did not translate into increased USC referrals or clinical outcomes changes. Short campaign duration and follow-up, and the small number of new lung cancer cases observed may have hampered detection effects.

## Background

Lung cancer has the highest mortality of all cancer types, accounting for a fifth of all cancer deaths worldwide.^1^ Lung cancer survival outcomes in the UK are amongst the worst of comparable high-income countries.^2^ High incidence and poor long-term survival mean that mortality rates are high,^3^ particularly in areas of high socioeconomic deprivation. Lung cancer incidence rises steeply with increasing deprivation and the survival inequality gap is widening.^4^ Delayed symptomatic presentation to a primary care physician and delays within and across primary and secondary care contribute to diagnosis of lung cancer in the later stages of disease.^5–7^ The possibility of curative treatment decreases with later-stage disease diagnosis; in the UK, less than a fifth of patients are eligible for surgical resection, in turn influencing outcomes.^6,8^

In the absence of routine lung cancer screening, early lung cancer diagnosis relies on prompt patient presentation and GP referral with symptoms indicative of lung cancer. Low public awareness of lung cancer symptoms is one possible barrier to prompt symptomatic presentation, contributing to normalisation and delay in reporting symptoms.^9–13^ Interventions are therefore required to raise lung cancer symptom awareness and reduce barriers to early presentation to expedite diagnosis.^14^ However, evidence of the impact of symptom awareness campaigns is limited.^15^ In 2008, the National Awareness and Early Diagnosis Initiative was formed in England with the aim of improving cancer outcomes through earlier diagnosis.^16^ Consequently, a national programme of cancer awareness activity was developed, including a focus on lung cancer, and there were also pockets of local activity. Evaluations of these interventions have suggested that these activities might have increased the number of lung cancers diagnosed at an earlier stage.^17–19^ However, to date, there is little evidence regarding the effectiveness of mass-media symptom awareness campaigns^18^ that have been adapted and implemented in different geographical settings and demographic contexts.

In response to poor lung cancer outcomes in Wales, and with evidence from the English Be Clear on Cancer (BCOC) campaign suggesting stage shift,^18^ the Welsh BCOC mass-media campaign was launched in 2016. The campaign was designed to increase public awareness of cough as a symptom of cancer and encourage adults over 50 years of age, especially among lower socioeconomic groups, to visit their GP if they had cough symptoms for three weeks or more. Cough is the most common presenting lung cancer symptom, and with reported low public awareness of cough as a cancer symptom, persistent cough was selected as the primary target symptom for the campaign.^9,20–24^

To evaluate the impact of the Welsh BCOC campaign, we conducted a quasi-experimental study with a before- and after- design, utilising survey responses and routinely collected data. Population based survey data to assess lung cancer symptom awareness and worry about wasting GP time as a perceived barrier to medical help seeking were compared one month before the campaign (pre-campaign period; June 2016) to one month after the campaign (post-campaign period; September 2016). Routine data including symptomatic presentations, primary care referrals, diagnostic testing and cancer diagnoses were compared in the campaign time period (July–August 2016) to the equivalent time period in 2015 (control period).

## Methods

The STROBE statement (strengthening the reporting of observational studies in epidemiology) guided reporting (Supplementary File 1).

### Intervention

To inform the campaign, six focus groups (four groups of current or former smokers, two groups of never smokers, total *n* = 48 participants) were undertaken in Wales during August 2015 to gauge audience receptiveness to existing lung cancer campaign materials from England, Scotland and Northern Ireland, who had already run national public awareness campaigns. Following minor adaptations from the English campaign, the Welsh lung cancer awareness campaign was launched in 2016 with the strapline “*If you’ve had a cough for three weeks or more, tell your doctor*” in both Welsh and English (Supplementary Files 2–5; https://www.cancerresearchuk.org/health-professional/awareness-and-prevention/be-clear-on-cancer/lung-cancer-awareness-campaign-wales).

Campaign messages were disseminated between 11 July and 11 August 2016 on television (S4C and ITV), online, bus and radio adverts, and on posters in pharmacies and on buses. The TV advert was intentionally crafted to appeal to older adults and people from lower socioeconomic (C2DE) groups. Where possible, campaign elements were targeted to reach more deprived groups (i.e. television scheduling, locations for advertising). Primary care engagement in advance of the campaign included circulating health care professional briefing materials with details of the campaign to health boards and primary care networks (https://www.cancerresearchuk.org/health-professional/awareness-and-prevention/be-clear-on-cancer/lung-cancer-awareness-campaign-wales#BCOC_Lung_Wales_Essential0). The first national BCOC respiratory campaign ran in England during the same time period as the Welsh lung BCOC campaign (July–October 2016). This also included three-week cough messaging utilised in previous English lung cancer campaigns, which was promoted via a range of channels including digital television with reach into Wales. This alignment of campaign timing aimed to increase the dissemination of the three-week cough message in Wales. English BCOC lung cancer campaigns that had run prior to 2016 had also utilised media channels that had reached into Wales, providing opportunity for Welsh audiences to have heard the three-week cough message in advance of the dedicated Welsh national activity.

## Participants and procedures

### Survey data

Pre-campaign (June 2016) and post-campaign (September 2016) population samples were surveyed by Beaufort Research, a market research company. Survey questions were informed by the Cancer Awareness Measure^25^ and previous campaign evaluation tracking in England, and were included as part of an omnibus survey carried out with a representative sample of the Welsh population (aged 16+) using face-to-face interviews.

Survey questions included gender, age, social group, number of children in the household and area of residence (Cardiff and South East Wales, Mid/West Wales, North Wales, Valleys and West/South Wales). Each respondent was allocated to ‘ABC1’ or ‘C2DE’ based on their responses to a range of profiling questions for social group, with ABC1 reflecting less deprived, and C2DE reflecting more deprived groups. Lung symptom awareness was measured using recall (“*There are many warning signs and symptoms of lung cancer. Please name as many as you are aware of”)* and prompted recognition questions (“*I’m now going to list some symptoms that may or may not be warning signs for lung cancer. For each one, can you tell me the extent to which you think it is a warning sign for lung cancer?”)*. For the recognition question, the symptoms listed were: *a cough for three weeks or more that does not go away, breathlessness, coughing up blood, a persistent pain in your chest or shoulder, losing weight for no obvious reason and a cough that got worse or changes*. Response options were: *definitely a warning sign, probably a warning sign, probably not a warning sign, definitely not a warning sign* and *don’t know*. Responses were dichotomised as definitely a warning sign/probably a warning sign versus definitely not a warning sign/probably not a warning sign. ‘Don’t know’ responses were not included in the regression analysis. To align with the focus of the campaign, data are reported for recognition/recall of cough symptom.

Worry about wasting the doctor’s time was measured using the question *“I’m going to read you a statement that is sometimes made about lung cancer. Can you please tell me how much you agree or disagree with it – that is whether you strongly agree, agree, disagree or strongly disagree. If I had a cough, I would be worried about wasting the GP/doctor’s time”*. Response options were dichotomised as strongly agree/agree versus strongly disagree/disagree.

### Routine data

Relevant routine data were sourced for pre-campaign (May and June), campaign (July and August) and post-campaign (September and October) periods in 2016, and the same periods in 2015. For the main comparison, the campaign period (July and August 2016) was compared to the comparable time period in the previous year (control period; July and August 2015). Health care and clinical outcomes included:

#### Primary care presentations

Anonymised counts of visits to GP practices for the cough symptom were identified using defined Read codes extracted from the Securely Anonymised Information Linkage (SAIL) databank. Counts were extracted for any patient registered with a GP practice in Wales using GP data within SAIL for all time points. The number of visits per practice/per week for the target cough symptom and control symptoms including neck pain, knee pain, shoulder pain and urinary tract infections in people aged 50 years and older was calculated for all time points.

#### Urgent suspected cancer (USC) referrals

USC referrals (patients with suspected lung cancer who are urgently referred from primary care to a specialist, and who are confirmed as urgent by the specialist) were received from the Welsh Government both at all-Wales and local health board (hospital) level. Conversion rates were calculated using the USC referral data received from Welsh Government. To calculate the conversion rate (the proportion of urgent referrals that resulted in a lung cancer diagnosis), the number of lung cancers diagnosed as a result of an urgent suspected cancer referral was divided by the number of USC referrals.

#### Radiology requests

The number of chest X-rays and chest CT scans carried out in each month (including tests with or without abdomen) was extracted for each health board from their systems. The aggregated data for Wales were used to calculate a count for all time points split by GP-referred and ‘all-referred’ tests. GP-referred tests were adjusted for working days because most GP surgeries are open Monday-Friday, while ‘all-referred’ tests could include referrals from other pathways that can occur on weekends.

#### Number of new lung cancers diagnosed, stage, source of referral

Lung cancer (ICD10 codes C33-C34) incidence data for non-small cell and small cell were extracted from the Cancer Network Information System Cymru (CaNISC) electronic patient records and split by month and year. Non-small cell lung cancer cases included histological, cytological or clinical diagnoses. The number of lung cancer cases diagnosed for the 2016 time points was compared to the number of cases diagnosed in the corresponding time periods in 2015. Staging data for small cell and non-small cell lung cancers were combined and grouped into early stage (stages I and II) and late stage (III and IV). The proportion of early and late stage cancers for known stages were calculated. The source of referral was extracted for patients diagnosed with lung cancer for the 2016 and 2015 time periods. Numbers and percentages of patients diagnosed with lung cancer following emergency attendance, accident and emergency admission, GP referral and consultant referral were identified from the CaNISC records. Diagnosis following referral after emergency attendance and accident and emergency admission were combined to ‘emergency department referral’.

#### First treatment received and performance status

The number of lung cancer cases in each treatment category were extracted for the 2016 time periods and the corresponding time periods in 2015. The proportion of all lung cancer cases by treatment group was compared across the 2015 and 2016 time points. Performance status data for all lung cancer cases diagnosed in the corresponding 2015 and 2016 time periods was extracted, according to the following categories: 0 (able to carry out normal activity without restriction), 1 (restricted in physically strenuous activity but ambulatory and able to carry out light work), 2 (ambulatory and capable of all self-care but unable to carry out any work up to about >50% of waking hours), 3 (capable of only limited self-care, confined to bed or chair >50% of waking hours), 4 (completely disabled, cannot carry out any self-care, totally confined to bed or chair) and unknown. Proportions of lung cancer cases by performance status category were compared across the 2015 control time period and 2016 campaign period.

### Statistical analysis

Multivariable logistic regression was used to investigate pre- to post- campaign differences in cough recall/recognition and worry about wasting the GP’s time, adjusting for demographic differences between survey samples. Survey data were weighted by age group within gender within Local Authority grouping, to be representative of the Welsh population. Interaction terms were used to test differences between social groups in awareness over time.

For the main comparison of the campaign period (July–August 2016) and the equivalent control period in the previous year (July–August 2015), changes in clinical outcomes were assessed using the two-sample test of proportions or the likelihood ratio tests for counts. The number of days was adjusted due to differences in the number of GP working days in each time period. The significance level was set at *p* < 0.001 to adjust for multiple testing.

## Results

### Survey sample characteristics

Sample characteristics are presented in Table 1. The pre-campaign (*n* = 1011) and post-campaign samples (*n* = 1013) were primarily female (pre-campaign 56.7%; post-campaign 53.1%), from the most deprived social group C2DE (pre-campaign 54.6%; post-campaign 53.8%), aged 45 years or over (pre-campaign 59.4%; post-campaign 60.7%) and resident in Cardiff/South East Wales (pre-campaign 26.2%; post-campaign 26.5%).

**Table 1:** Demographic characteristics of survey sample pre- and post-campaign.

	Pre-campaign (June 2016; *n* = 1011)		Post-campaign (Sept 2016; *n* = 1013)	
	*N*	%	*N*	%
Gender				
Male	438	43.3	475	46.9
Female	573	56.7	538	53.1
Age				
16–24	127	12.6	89	8.8
25–34	154	15.2	168	16.6
35–44	130	12.9	141	13.9
45–54	174	17.2	153	15.1
55–64	138	13.7	157	15.5
65 and over	288	28.5	305	30.1
Social group (SES)				
AB (most affluent)	144	14.2	176	17.4
C1	315	31.2	291	28.7
C2	194	19.2	188	18.6
DE (most deprived)	358	35.4	356	35.2
Area of residence				
Cardiff/South East Wales	265	26.2	268	26.5
Mid/West Wales	162	16.0	166	16.4
North Wales	208	20.6	230	22.7
Valleys	174	17.2	181	17.9
West/South Wales	202	20.0	168	16.6
Children in household				
Yes	323	31.9	344	34
No	688	68.1	669	66

### Cough symptom awareness and worry about wasting the doctor’s time

As shown in Table 2, there was a statistically significant 13.3% increase in recall (*p* < 0.001) and 4.4% increase in recognition (*p* < 0.001) of the cough symptom pre- to post-campaign. There was a statistically significant 7.5% increase in recall of shortness of breath (*p* < 0.001) and 11.7% reduction in the number of people who could not recall any symptoms of lung cancer (*p* < 0.001) pre-to-post campaign. The relationship between social group and recognition of the cough symptom was not significant (*p* = 0.370 for the interaction term).

**Table 2:** Public awareness and barriers to help seeking pre- and post-campaign survey results.

Survey question	Pre-campaign (June 2016; *n* = 1011)	Post-campaign (Sept 2016; *n* = 1013)	% change	*p*-value
Symptom recall				
Cough/coughing	27.5%	40.8%	**13.3%**	**<0.001**
Persistent/ long lasting/ bad cough	14.2%	15.4%	1.2%	0.26
Shortness of breath/ bad chest/difficulty breathing	29.7%	37.2%	**7.5%**	**<0.001**
Coughing up blood/ blood in mouth or mucus	22.5%	18.1%	−4.4%	0.05
Do not know/none	31.4%	19.7%	**−11.7%**	**<0.001**
Symptom recognition				
A cough for three weeks or more that does not go away	82.2%	86.6%	**4.4%**	**<0.001**
Breathlessness	83.6%	85.9%	2.3%	0.39
Coughing up blood	95.5%	93.3%	−2.2%	0.02
A persistent pain in your chest or shoulder	68.9%	67.2%	−1.7%	0.18
Losing weight for no obvious reason	78.5%	74.7%	−3.8%	0.02
A cough that has got worse or changes	87.1%	88.9%	1.8%	0.32
Recognition of three-week cough by social group				
ABC1 (most affluent)	80.5%	83.9%	3.4%	0.16
C2DE (most deprived)	86.3%	87.2%	0.9%	0.02
Worry about wasting GP time				
ABC1 (most affluent)	47.9%	46.3%	−1.6%	0.33
C2DE (most deprived)	50.1%	40.1%	**−10.0%**	**<0.001**
Total	49.2%	42.8%	**−6.4%**	**<0.001**

Results in bold indicate a statistically significant change between 2015 and 2016

There was a statistically significant 6.4% reduction in worry about wasting the GP’s time pre- to post-campaign (*p* < 0.001). There was a statistically significant 10% decline in worry about wasting GP time for C2DE (the most deprived group) pre- to post-campaign (*p* = 0.001), but a non-statistically significant 1.6% decline for ABC1 (the more affluent group) (*p* = 0.33).

### GP presentations

The number of GP visits for cough symptoms increased significantly between the control and campaign period in all age groups apart from 10–19-year olds (Table 3). The total number of people of all ages presenting to their GP with a cough during the 2016 campaign period increased significantly by 21.4% (*p* < 0.001) compared to the corresponding control period in 2015. In the target age group of people aged 50 years and over, there was a statistically significant 24.3% increase (*p* < 0.001) in the number of visits to a GP for cough during 2016 compared to the equivalent 2015 control period.

**Table 3:** Number of cough presentations adjusted weekly rate of GP presentations for cough and four control symptoms in patients aged 50 years and cough presentations by age group.

	2015; *n*	2016; *n*	Change (*n*)	% change	*P*-value
*Number of presentations per practice per week in patients over the age of 50 (adjusted*^a^*)*					
Cough					
Pre-campaign	7.9	7.6	−0.3	−3.1	
Campaign	6.0	7.7	1.7	**28.9**	**<0.001**
Post-campaign	8.1	8.3	0.2	2.1	
Urinary tract infection					
Pre-campaign	1.0	1.0	0.0	1.1	
Campaign	1.0	1.1	0.1	4.5	0.77
Post-campaign	1.1	1.1	0.1	6.3	
Neck pain					
Pre-campaign	1.0	1.1	0.1	3.0	
Campaign	1.0	1.0	0.0	0.7	0.26
Post-campaign	1.1	1.1	0.0	−2.2	
Shoulder pain					
Pre-campaign	0.1	0.1	0.0	5.9	
Campaign	0.1	0.1	0.0	14.7	0.23
Post-campaign	0.1	0.1	0.0	0.8	
Knee pain					
Pre-campaign	3.2	3.2	0.0	2.1	
Campaign	2.9	3.1	0.2	5.1	0.37
Post-campaign	2.9	3.0	0.1	1.7	
*Number of presentations for cough symptom during the campaign period (July and August 2016) and corresponding time period in the previous year (July and* *August 2015) by age group*					
Age group					
0–9	5745	6612	867	**15.1**	**<0.001**
10–19	1870	2061	191	10.2	0.002
20–29	2112	2561	449	**21.3**	**<0.001**
30–39	2155	2747	592	**27.5**	**<0.001**
40–49	3075	3673	598	**19.4**	**<0.001**
50–59	4197	5324	1127	**26.9**	**<0.001**
60–69	5374	6605	1231	**22.9**	**<0.001**
70–79	4676	5900	1224	**26.2**	**<0.001**
80+	3050	3678	628	**20.6**	**<0.001**
Total aged 50+	17,297	21,507	4210	**24.3**	**<0.001**
Total all ages	32,254	39,161	6907	**21.4**	**<0.001**

Results in bold indicate a statistically significant change between 2015 and 2016

^a^Adjusted for working days

Among the target over 50 s age group, the number of presentations with a cough increased from 6.0 per GP practice per week during 2015 to 7.7 per GP practice per week during the campaign period in 2016, equivalent to a statistically significant increase of 28.9% (*p* < 0.001). In the same time period, there was no significant increase in the number of GP presentations among the over 50 age group for each of the four control symptoms of urinary tract infection (*p* = 0.77), neck pain (*p* = 0.26), shoulder pain (*p* = 0.23) or knee pain (*p* = 0.37) (Table 3).

### Urgent suspected cancer referrals and conversion rate

There was a non-significant 1.2% reduction (*p* = 0.82) in the total number of USC referrals for suspected lung cancer, and a non-significant 1.4% reduction (*p* = 0.56) in the number of USC referrals resulting in a lung cancer diagnosis (conversion rate) between the 2016 campaign period and the equivalent time period in 2015 (Table 4).

**Table 4:** Urgent suspected lung cancer referrals and conversion rate.

	2015	2016	% change (adjusted^a^)	*P*-value
Urgent suspected lung cancer referrals (total, *n*)				
Pre-campaign	623	659	3.3	
Campaign	650	642	−1.2	0.82
Post-campaign	581	559	−1.6	
Conversion rate (%)				
Pre-campaign	24.2%	24.9%	0.6	
Campaign	25.1%	23.7%	−1.4%	0.56
Post-campaign	22.9%	23.1%	0.2%	

^a^Adjusted for working days

### Radiology requests

A statistically significant increase of 23.4% in GP-referred chest X-rays (*p* < 0.001), and 8.1% increase in chest X-rays from all referral sources (*p* < 0.001) was reported in the 2016 campaign period compared to the equivalent time period in 2015 (Table 5).

**Table 5:** Number of chest x-rays and chest CT scans conducted.

	Number of tests (*n*)		Tests per day (adjusted^a^)			
	2015	2016	2015	2016	% change	*P*-value
GP-referred chest X-rays						
Pre-campaign	21714	23092	529.6	549.8	3.8	
Campaign	19107	23585	444.3	548.5	**23.4**	**<0.001**
Post-campaign	21368	23409	485.6	544.4	12.1	
All chest X-rays						
Pre-campaign	77763	79787	1896.7	1899.7	0.2	
Campaign	73690	79686	1713.7	1853.2	**8.1**	**<0.001**
Post-campaign	77556	80912	1762.6	1881.7	6.8	
GP-referred chest CT scans						
Pre-campaign	746	843	18.2	20.1	10.3	
Campaign	780	855	18.1	19.9	9.6	0.06
Post-campaign	678	791	15.4	18.4	19.4	
All Chest CT scans						
Pre-campaign	5207	6040	127	143.8	13.2	
Campaign	5244	5775	122	134.3	**10.1**	**<0.001**
Post-campaign	5390	5871	122.5	136.5	11.5	

Results in bold indicate a statistically significant change between 2015 and 2016

^a^Adjusted for working days

There was a non-statistically significant 9.6% increase in GP-referred chest CT scans in the 2016 campaign period compared to the equivalent time period in 2015 (*p* = 0.06). There was a statistically significant 10.1% increase in the number of chest CT scans from all referral sources (*p* < 0.001) in the 2016 campaign period compared to the equivalent time period in 2015 (Table 5).

### Number and stage of new lung cancers diagnosed

There were no statistically significant changes in the number of new diagnoses of non-small cell lung cancer (*p* = 0.34), small cell lung cancer (*p* = 0.38) or the total number of new lung cancer diagnoses (*p* = 0.70) in the 2016 campaign period compared to the equivalent time period in 2015 (Table 6).

**Table 6:** Number and stage of lung cancer patients, with diagnosis by source of referral.

	2015 Number of cases (%)	2016 Number of cases (%)	n change	*p*-value
*Number of new lung cancer cases*				
Non-small cell lung cancer				
Pre-campaign	324	326	2	
Campaign	348	372	24	0.34
Post-campaign	342	318	−24	
Small-cell lung cancer				
Pre-campaign	51	47	−4	
Campaign	53	40	−13	0.38
Post-campaign	42	39	−3	
Total				
Pre-campaign	375	373	−2	
Campaign	401	412	11	0.70
Post-campaign	384	357	−27	

	2015 Number of cases (%)	2016 Number of cases (%)	% Change	*p*-value
*Stage of new lung cancer cases number and proportion of cases with known stage*^a^				
Early (stage I and II)				
Pre-campaign	95 (25.5%)	85 (23.4%)	−2.1%	
Campaign	98 (24.8%)	114 (28.3%)	3.5%	0.27
Post-campaign	114 (30.2%)	94 (27.0%)	−3.2%	
Late (stage III and IV)				
Pre-campaign	277 (74.5%)	278 (76.6%)	2.1%	
Campaign	297 (75.1%)	289 (71.7%)	−3.5%	0.27
Post-campaign	263 (69.8%)	254 (73.0%)	3.2%	
Stage unknown				
Pre-campaign	3	10		
Campaign^b^	6	9		
Post-campaign	7	9		
*Number and proportion of new lung cancer cases diagnosed by source of referral*				
Following A&E attendance or emergency admission				
Pre-campaign	102 (27.2%)	111 (29.8%)	2.6%	
Campaign	89 (22.2%)	117 (28.5%)	6.3%	0.04
Post-campaign	103 (26.8%)	108 (30.3%)	3.4%	
Referral from GP				
Pre-campaign	176 (46.9%)	184 (49.3%)	2.4%	
Campaign	180 (44.9%)	171 (41.6%)	−3.3%	0.35
Post-campaign	154 (40.1%)	146 (40.9%)	0.8%	
Referral from a consultant (other than in an A&E department)				
Pre-campaign	73 (19.5%)	57 (15.3%)	−4.2%	
Campaign	112 (27.9%)	85 (20.7%)	−7.2%	0.02
Post−campaign	103 (26.8%)	77 (21.6%)	−5.3%	
Referral from a consultant (from inpatients)				
Pre-campaign	13 (3.5%)	9 (2.4%)	−1.1%	
Campaign	16 (4.0%)	24 (5.8%)	1.8%	0.22
Post-campaign	11 (2.9%)	16 (4.5%)	1.6%	
Other source				
Pre-campaign	11 (2.9%)	12 (3.2%)	0.3%	
Campaign	4 (1.0%)	14 (3.4%)	2.4%	0.02
Post-campaign	13 (3.4%)	10 (2.8%)	−0.6%	
Not recorded				
Pre-campaign	0 (0%)	0 (0%)	0%	
Campaign	0 (0%)	1 (0.2%)	0.2%	
Post-campaign	0 (0%)	0 (0%)	0%	

^a^Small-cell and non-small cell lung cancer cases combined; %’s presented as proportions of the total known cases

^b^Statistical testing not conducted due to very small numbers

There were no statistically significant differences in staging data. There was a non-statistically significant 3.5% increase in the total number of early stage (I and II) cases of lung cancer cases (*p* = 0.27) and a 3.5% non-significant decrease in the total number of late stage (III and IV) cases of lung cancer (*p* = 0.27) in the 2016 campaign period compared to the equivalent time period in 2015 (Table 6).

### Referral source of number of lung cancers diagnosed

There were no statistically significant changes in the number of new lung cancer diagnoses from all referral sources. There was a non-statistically significant increase in the proportion of new lung cancer diagnoses after referral through emergency department (6.3% increase; *p* = 0.04), referral through from an inpatient consultant (1.8% increase; *p* = 0.22) and referral from other sources (2.4% increase; *p* = 0.02) during the 2016 campaign to the equivalent time period in 2015 (Table 6).

There was a non-statistically significant decrease in the proportion of new lung cancer diagnoses after referral through from a non-accident and emergency department consultant (7.2% decrease; *p* = 0.02), and referral from the GP (3.3% decrease; *p* = 0.35) during the 2016 campaign period to the equivalent time period in 2015 (Table 6).

### First treatment received and performance status of lung cancers diagnosed

For all forms of treatment including surgical resection, there was no statistically significant difference during the 2016 campaign period to the equivalent time period in 2015 (Supplementary File 6). There were no statistically significant changes in all performance status categories for new lung cancer patients during the 2016 campaign to the equivalent time period in 2015 (Supplementary File 7).

## Discussion

We evaluated the impact of the first nationwide mass-media lung cancer symptom awareness campaign to be conducted in Wales. The campaign was successful in raising public awareness of cough as a symptom of lung cancer and in reducing barriers to symptomatic presentation. A greater reduction in worry about wasting GP time was observed after the campaign, especially among socioeconomically deprived groups. Behavioural changes were observed during the campaign, with an increase in the number of patients presenting to their GP with a cough symptom for the target over 50 s target group. Although GP-ordered chest X-rays increased during the campaign, this did not extend to USC referrals or the number and stage distribution of new lung cancer diagnoses.

The first national BCOC lung cancer campaign in England, involving a four-week regional pilot in the central TV region in 2011 and eight-week national mass-media campaign in 2012, reported increased symptom awareness, primary care cough symptom presentations and GP requested chest X-rays.^17,18^ A subsequent community-based lung cancer awareness campaign run over an extended period of time, combining public awareness activities with open-access walk-in chest X-ray for those with symptoms that could indicate lung cancer and GP education^19^ also reported improved lung cancer outcomes including a highly significant stage shift and higher treatment rates. We found a significant increase in the number of GP presentations with a cough symptom and GP-ordered chest X-ray requests. However, when comparing the campaign time period in 2015 to the pre- and post-campaign time period data for 2015 (Tables 3 and 5), the reported number of cough symptom presentations and GP-ordered chest X-rays are substantially lower. The lower number of cough presentations and radiology requests in the 2015 campaign period may either be lower by chance (thus artificially inflating our findings), or reflect the time of year when the 2016 campaign was run (July–August) outside of flu season.

We found no impact of the Welsh BCOC campaign on USC referrals or other clinical outcomes. Our findings likely reflect the need for higher-intensity briefings for health-professionals with information about campaign activities and symptoms for referral. Further, it is possible that despite the increase in cough presentations, system and access barriers from primary care to secondary care in Wales may have led to no increase in the proportion of lung cancer cases diagnosed through the GP referral route, reflecting the need for service redesign.

The current campaign was designed to target adults over the age of 50, particularly from C2DE audiences. However, public-facing materials did not display age-related risk information to maintain simple campaign messaging. Campaign developers selected actors for the campaign materials to implicitly reinforce age. It is possible that without explicit advice on age-related risk, younger and lower risk individuals presented to primary care with symptoms, impacting USC referrals. We report the largest reduction in worry about wasting GP time as a psychosocial barrier to help seeking in the target deprived group. Our findings may reflect successful strategic targeting of campaign messages to areas of high socioeconomic deprivation in Wales to modify salient barriers to help seeking.

Low campaign dose and intensity limited the impact of the Welsh BCOC campaign on health care activity and clinical outcomes. Additionally, possible contamination from previous English Be Clear on Cancer lung campaigns into Wales (principally via digital channels) may have potentially diluted the effect of the Welsh Be Clear on Cancer campaign because people in Wales may have previously been exposed to campaign messaging. Due to funding constraints, the dedicated Welsh campaign materials were delivered through fewer outlets and with lower intensity, and the duration of the campaign was half the dose of the first eight-week English BCOC campaign.^17,18^ Longer campaign duration, together with a more comprehensive and multi-faceted mode of delivery, may lead to larger effects, for example the five-year community-based Leeds lung cancer campaign reported an increase of 80% in chest X-ray referrals.^19^

The methodological limitations associated with the evaluation of cancer awareness campaigns may also explain these findings.^16^ Small numbers of new cases during the campaign and comparison periods hampered effect detection for new/early stage lung cancer diagnoses. Due to funding limitations and the time-sensitive nature of the project, it is possible that the follow-up period restricted the capture of changes to clinical outcomes and is a limitation of the evaluation. A long follow-up period is required to account for the time lag between campaign implementation and radiology/suspected cancer referral, and to collect clinical data for patients presenting with symptoms during the campaign who were subsequently diagnosed with lung cancer. Further, data were obtained from two sources, including Omnibus surveys and routinely collected data records. Variation in data collection time points precluded direct comparison of data at each time point. Future campaign evaluations could assess the possible negative effects of the campaign, such as increased health anxiety.

Promisingly, our findings show that a mass-media cough campaign can increase symptom awareness, symptomatic presentation and, potentially, GP-ordered diagnostic testing. We found evidence of reach and reduced barriers to help seeking in socioeconomically deprived groups.

## Conclusion

Increased public awareness, cough symptom presentation and GP-ordered diagnostic testing did not translate into increased USC referrals, new lung cancer diagnoses or stage shift following a national mass-media lung cancer awareness campaign in Wales. This reflected limitations of campaign delivery and methodological issues associated with its evaluation. Earlier diagnosis might be achieved by more intensive, sustained and targeted campaigns, by improving GP diagnostic and referral systems, and through secondary care pathway redesign.

## Supplementary information

Suppl. Files merged

## Data Availability

Requests for data-sharing will be considered by the senior authors. Please submit requests to the corresponding author.
