# Supplementary material for: Evaluation of a national lung cancer symptom awareness campaign in Wales
Source: Br J Cancer. 2019 Dec 16;122(4):491–7. doi: 10.1038/s41416-019-0676-2 (PMC7029011; doi:10.1038/s41416-019-0676-2)
Supplement: Supplementary file 1 — Suppl. Files merged [file 41416_2019_676_MOESM1_ESM.pdf]

STROBE Statement—checklist of items that should be included in reports of observational studies

	Item No	Recommendation	Page number
**Title and abstract**	1	(*a*) Indicate the study's design with a commonly used term in the title or the abstract	1
		(*b*) Provide in the abstract an informative and balanced summary of what was done and what was found	2
**Introduction**			
Background/rationale	2	Explain the scientific background and rationale for the investigation being reported	3
Objectives	3	State specific objectives, including any prespecified hypotheses	3
**Methods**			
Study design	4	Present key elements of study design early in the paper	3-5
Setting	5	Describe the setting, locations, and relevant dates, including periods of recruitment, exposure, follow-up, and data collection	3-5
Participants	6	(*a*) *Cohort study*—Give the eligibility criteria, and the sources and methods of selection of participants. Describe methods of follow-up<br>*Case-control study*—Give the eligibility criteria, and the sources and methods of case ascertainment and control selection. Give the rationale for the choice of cases and controls<br>*Cross-sectional study*—Give the eligibility criteria, and the sources and methods of selection of participants	4-5
		(*b*) *Cohort study*—For matched studies, give matching criteria and number of exposed and unexposed<br>*Case-control study*—For matched studies, give matching criteria and the number of controls per case	
Variables	7	Clearly define all outcomes, exposures, predictors, potential confounders, and effect modifiers. Give diagnostic criteria, if applicable	4-6
Data sources/ measurement	8*	For each variable of interest, give sources of data and details of methods of assessment (measurement). Describe comparability of assessment methods if there is more than one group	4-6
Bias	9	Describe any efforts to address potential sources of bias	5-6
Study size	10	Explain how the study size was arrived at	N/A
Quantitative variables	11	Explain how quantitative variables were handled in the analyses. If applicable, describe which groupings were chosen and why	6
Statistical methods	12	(*a*) Describe all statistical methods, including those used to control for confounding	6
		(*b*) Describe any methods used to examine subgroups and interactions	6
		(*c*) Explain how missing data were addressed	6
		(*d*) *Cohort study*—If applicable, explain how loss to follow-up was addressed<br>*Case-control study*—If applicable, explain how matching of cases and controls was addressed<br>*Cross-sectional study*—If applicable, describe analytical methods taking account of sampling strategy	N/A
		(*e*) Describe any sensitivity analyses	6

**Results**			**Page number**
Participants	13*	(a) Report numbers of individuals at each stage of study—eg numbers potentially eligible, examined for eligibility, confirmed eligible, included in the study, completing follow-up, and analysed	6
		(b) Give reasons for non-participation at each stage	N/A
		(c) Consider use of a flow diagram	N/A
Descriptive data	14*	(a) Give characteristics of study participants (eg demographic, clinical, social) and information on exposures and potential confounders	Table 1
		(b) Indicate number of participants with missing data for each variable of interest	N/A
Outcome data	15*	*Cohort study*—Report numbers of outcome events or summary measures over time<br>*Case-control study*—Report numbers in each exposure category, or summary measures of exposure<br>*Cross-sectional study*—Report numbers of outcome events or summary measures	7-9
Main results	16	(*a*) Give unadjusted estimates and, if applicable, confounder-adjusted estimates and their precision (eg, 95% confidence interval). Make clear which confounders were adjusted for and why they were included	7-9, Tables 3-6
		(*b*) Report category boundaries when continuous variables were categorized	7, Table 2
		(*c*) If relevant, consider translating estimates of relative risk into absolute risk for a meaningful time period	N/A
Other analyses	17	Report other analyses done—eg analyses of subgroups and interactions, and sensitivity analyses	6
**Discussion**			
Key results	18	Summarise key results with reference to study objectives	9
Limitations	19	Discuss limitations of the study, taking into account sources of potential bias or imprecision. Discuss both direction and magnitude of any potential bias	10
Interpretation	20	Give a cautious overall interpretation of results considering objectives, limitations, multiplicity of analyses, results from similar studies, and other relevant evidence	10-11
Generalisability	21	Discuss the generalisability (external validity) of the study results	10
**Other information**			
Funding	22	Give the source of funding and the role of the funders for the present study and, if applicable, for the original study on which the present article is based	11

*Give information separately for cases and controls in case-control studies and, if applicable, for exposed and unexposed groups in cohort and cross-sectional studies.

Note: An Explanation and Elaboration article discusses each checklist item and gives methodological background and published examples of transparent reporting. The STROBE checklist is best used in conjunction with this article (freely available on the Web sites of PLoS Medicine at http://www.plosmedicine.org/, Annals of Internal Medicine at http://www.annals.org/, and Epidemiology at http://www.epidem.com/). Information on the STROBE Initiative is available at www.strobe-statement.org.

GIG CYMRU NHS WALES

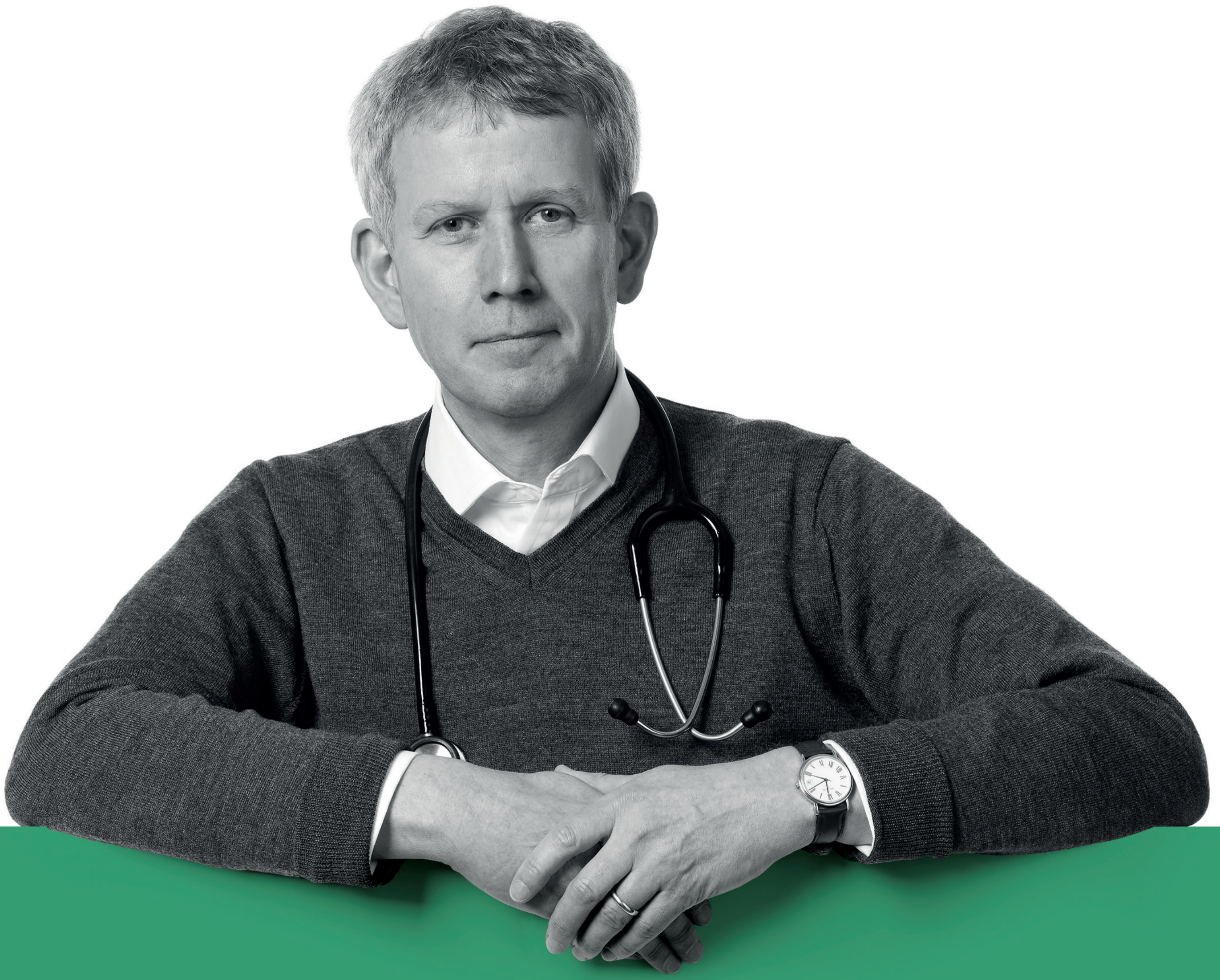

Dr Nick Davies

Wedi bod yn pesychu am 3 wythnos?

Dywedwch wrth eich meddyg.

Gallai peswch cyson fod yn arwydd o ganser yr ysgyfaint. Mae ei ganfod yn gynnar yn golygu ei fod yn haws ei drin.

BOD YN GLIR AM GANSER

ewch.gig.cymru/canseryrysgyfaint

Been coughing for 3 weeks?

Tell your doctor.

A persistent cough could be a sign of lung cancer. Finding it early makes it more treatable.

BE CLEAR ON CANCER

go.nhs.wales/lungcancer

CANCER RESEARCH UK

Call us free, 365 days a year
0808 808 1010
Ffoniwch ni, 365 diwrnod y flwyddyn

tenovus cancer care
gofal canser

Cefnogir gan Lywodraeth Cymru
Supported by Welsh Government

GIG CYMRU NHS WALES

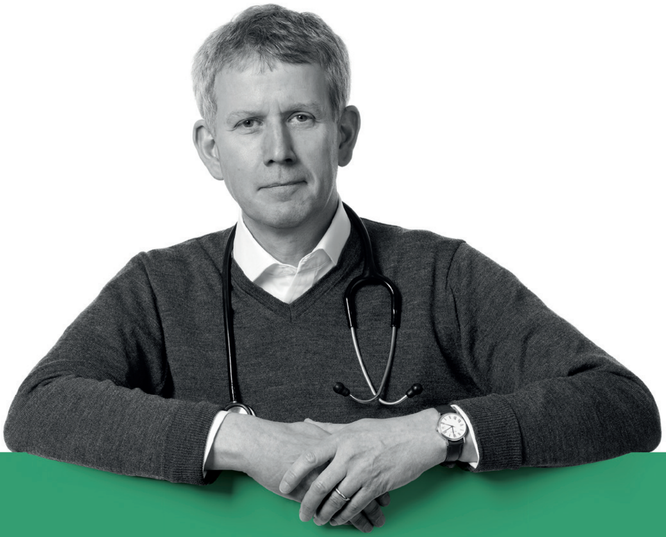

Dr Nick Davies

Wedi bod yn pesychu am 3 wythnos?

Dywedwch wrth eich meddyg.

BOD YN GLIR AM GANSER

ewch.gig.cymru/canseryrysgyfaint

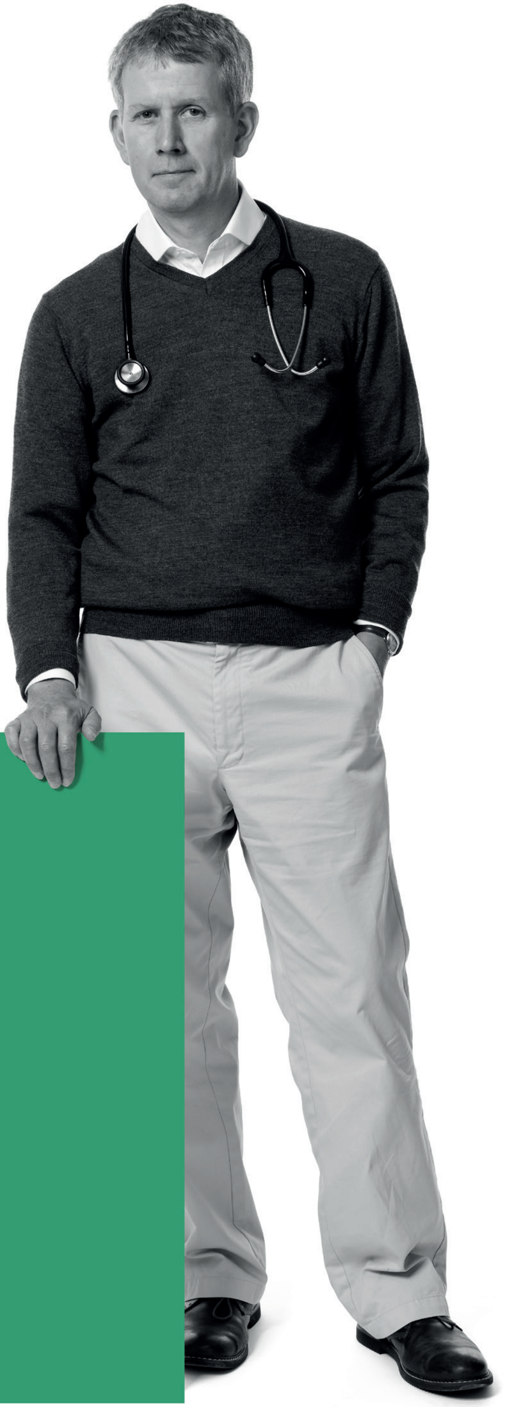

Dr Nick Davies

Rhaid bod yn glir…

Canser yr ysgyfaint yw un o'r canserau mwyaf cyffredin yng Nghymru.

Mae oddeutu 2,400 o achosion newydd yng Nghymru bob blwyddyn. Mae'n lladd mwy o ddynion a merched nag unrhyw fath arall o ganser.

…am ganser yr ysgyfaint

Gall canser yr ysgyfaint effeithio ar bobl o bob oedran ond mae'n dechrau dod yn fwy cyffredin ar ôl 50 oed. Er ei fod yn fwy cyffredin ymhlith pobl sy'n ysmygu, nid yw oddeutu un o bob wyth o bobl sydd â chanser yr ysgyfaint wedi ysmygu erioed.

Mae canfod canser yr ysgyfaint yn gynnar yn gwella'r siawns o driniaeth lwyddiannus.

Felly os oes gennych chi beswch ers tair wythnos a mwy, mae'n werth cysylltu â'ch meddyg er mwyn bod yn ddiogel.

...am sut mae ei ganfod

Mae'n rhaid i chi ddweud wrth eich meddyg ar unwaith os ydych chi wedi bod yn pesychu ers tair wythnos neu fwy. Dyma rai o symptomau eraill canser yr ysgyfaint:

- Peswch sydd wedi gwaethygu neu newid
- Haint dro ar ôl tro ar y frest
- Pesychu gwaed
- Colli eich gwynt
- Teimlo'n fwy blinedig nag arfer ers peth amser
- Colli pwysau am ddim rheswm amlwg
- Cur neu boen yn eich brest neu eich ysgwydd sydd wedi para am gryn amser.

Os ydych yn sylweddoli unrhyw o'r symptomau yma, cysylltwch â'ch meddyg yn syth.

...am ba mor bwysig yw dweud wrth eich meddyg

Os byddwch yn sylwi ar unrhyw rai o'r symptomau hyn, dywedwch wrth eich meddyg ar unwaith. Mae canfod canser yr ysgyfaint yn gynnar yn ei wneud yn haws ei drin felly gall dweud wrth eich meddyg ar unwaith achub eich bywyd. Mae'n bur debyg nad yw'n ddifrifol ond fe allai fod yn arwydd o rywbeth arall sydd angen ei drin.

Felly peidiwch ag anwybyddu'r symptomau na gohirio dweud wrth eich meddyg. Gall wneud byd o wahaniaeth a 'fyddwch chi ddim yn gwastraffu amser neb.

Ac os ydych chi'n gwybod am unrhyw un sydd â'r symptomau yma, mynnwch eu bod yn dweud wrth eu meddyg. Eto, mae'n bur debyg nad yw'n ddifrifol, ond dylent gael archwiliad.

...am siarad â'ch meddyg

Bydd eich meddyg yn gofyn ambell gwestiwn, fel y rhai isod, ac efallai y bydd yn awgrymu archwiliad pelydr-x ar eich brest. Mae hon yn drefn arferol a does dim angen poeni. Mae cael archwiliad pelydr-x yn syml a chyflym heb fod angen aros yn yr ysbyty dros nos.

Efallai y bydd eich meddyg yn gofyn rhai o'r cwestiynau hyn i chi:

- Ers faint mae'r peswch wedi bod gennych chi?
- Ydi eich peswch wedi newid gydag amser?
- Ydych chi wedi pesychu gwaed?
- Ydych chi wedi cael haint ar y frest yn ddiweddar?
- Ydych chi wedi bod yn fyr o wynt? Ers faint?
- Ydi sut rydych chi'n fyr eich gwynt wedi newid dros amser?
- Ydych chi wedi colli unrhyw bwysau?
- Ydych chi wedi cael poen yn eich brest neu eich ysgwydd?
- Ydi'r boen yma wedi newid gydag amser?

Efallai y bydd ysgrifennu eich symptomau a sut rydych yn teimlo yn helpu, rhag ofn i chi anghofio rhywbeth wrth ddweud wrth eich meddyg.

...am leihau eich risg o gael canser

Gallwch leihau'r risg drwy newid eich ffordd o fyw, gan gynnwys;

Rhoi'r gorau i ysmygu
Mae ysmygu yn cynyddu'r risg o sawl math o ganser. Os ydych yn ysmygu, y peth gorau y gallwch ei wneud ar gyfer eich iechyd yw rhoi'r gorau iddi. Mae digon o gefnogaeth ar gael gan y GIG yng Nghymru. Ewch i **dimsmygucymru.com** neu gallwch rhadffôn **0800 0852219**.

Edrych ar ôl eich hun
Gall bod dros bwysau neu'n ordew gynyddu eich risg o rai canserau. Ceisiwch gynnal pwysau iach a chadw yn weithgar. Nofio, beicio, dawnsio, cerdded – po fwyaf y byddwch yn ei wneud, y gorau. Ceisiwch fwyta diet iach, cytbwys hefyd, gyda digon o ffrwythau a llysiau.

Torrwch i lawr ar alcohol
Gall yfed gormod o alcohol arwain at nifer o broblemau iechyd ac mae'n gysylltiedig â rhai mathau o ganser. Drwy yfed llai byddwch yn lleihau eich risgiau iechyd.

... am sut gallai siarad â'ch meddyg yn gynnar achub eich bywyd chi

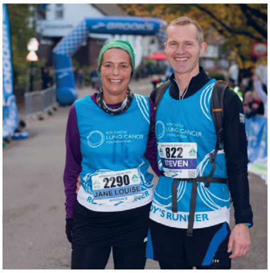

Jane Holmes

"Fe gefais i ddiagnosis o ganser yr ysgyfaint ym mis Mehefin 2014. Roeddwn i'n byw yn iach, yn rhedeg yn rheolaidd, yn bwyta'n iach a doeddwn i ddim yn ysmygu. Fel y gallwch chi ddychmygu, roedd y diagnosis a'r prognosis yn gryn dipyn o sioc.

Ers hynny rydw i wedi cael cemotherapi, radiotherapi ac wedyn roeddwn i'n gymwys i gael llawdriniaeth. Fe gefais i lawdriniaeth ym mis Mehefin 2015 a phedwar mis yn ddiweddarach fe wnes i gwblhau Marathon Eryri! Rydw i wedi cael sgans clir ers y llawdriniaeth ac rydw i wedi bod ar sawl gwyliau hyfryd ac wedi rhoi fy enw ar gyfer y marathon eto eleni!

Rydw i'n cael fy monitro'n rheolaidd ond yn falch iawn o fod yn gwneud mor dda."

"Felly dyma ble roeddwn i, saith mlynedd yn ddiweddarach, ar ôl rhoi'r gorau i ysmygu ar ôl gwneud hynny am 50 mlynedd. Doeddwn i ddim mewn peryg o gwbl, roeddwn i'n teimlo'n dda ac yn edrych ymlaen at weddill fy mywyd ar ôl ymddeol. Ym mis Chwefror 2014, heb unrhyw rybudd o gwbl, fe gefais i bwl o niwmonia ac ar ôl cael triniaeth i glirio hwnnw fe gefais i ddiagnosis o ganser yr ysgyfaint. Ddwy flynedd ar ôl cael y driniaeth, rydw i'n teimlo'n dda nawr ac, yn ôl yr hyn mae pobl yn ei ddweud wrtha i, yn edrych yn dda. Peidiwch byth â meddwl ''fydd o ddim yn digwydd i mi' a pheidiwch byth â meddwl 'mae gen i ofn gofyn am help'. Mae pobl wych ar gael i roi triniaethau a gofal gwych. 'Fyddwch chi ddim ar eich pen eich hun, rydych chi'n mynd drwy'r profiad gyda'ch gilydd."

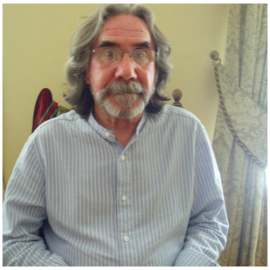

Graham Thomas

Aneglur am unrhyw beth?

Ewch i **ewch.gig.cymru/canseryrysgyfaint**

CANCER RESEARCH UK

Ffonio'n unig sydd angen
0808 808 1010
Ffoniwch ni, 365 diwrnod y flwyddyn

tenovus cancer care
gofal canser

Community Pharmacy Wales
Fferylliaeth Gymunedol Cymru

Cefnogir gan Lywodraeth Cymru
Supported by Welsh Government

GIG CYMRU NHS WALES

GIG CYMRU
NHS WALES

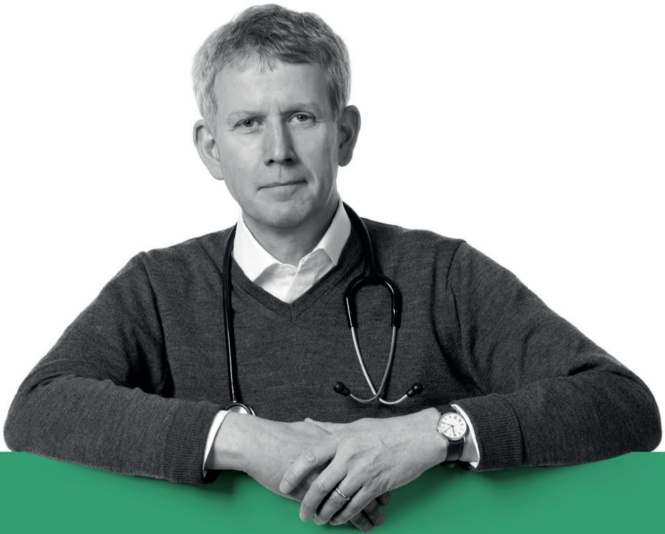

Dr Nick Davies

Been coughing for 3 weeks?

Tell your doctor.

BE CLEAR ON CANCER

go.nhs.wales/lungcancer

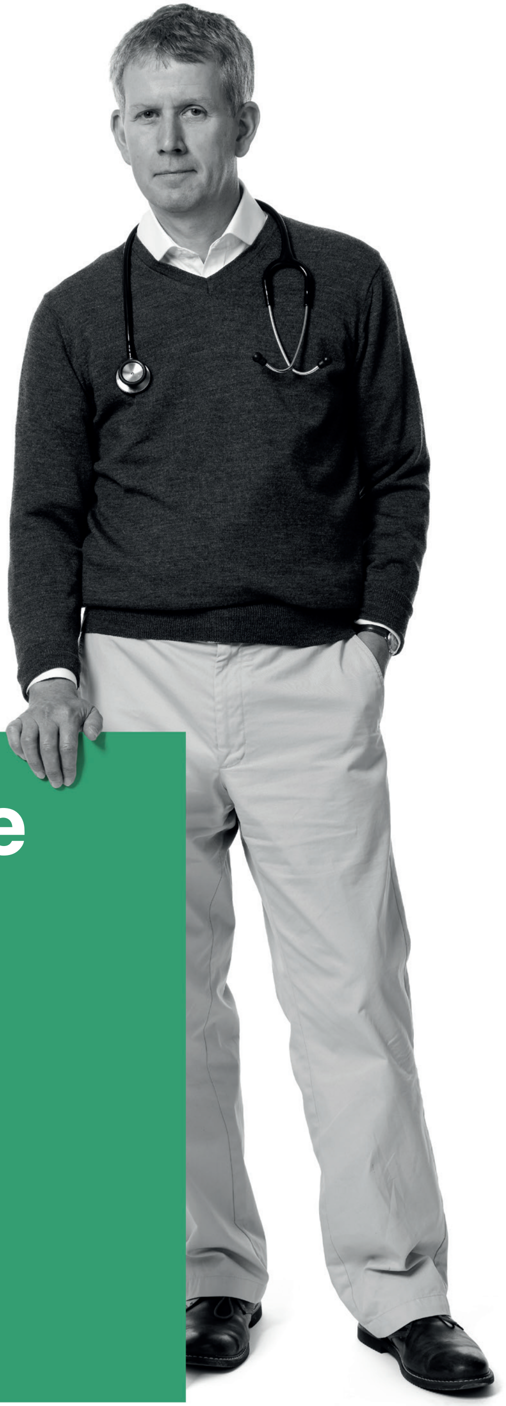

Dr Nick Davies

Let’s be clear…

Lung cancer is one of the most common cancers in Wales.

There are around 2,400 new cases in Wales every year. It kills more men and women than any other form of cancer.

…about lung cancer

Lung cancer can affect people of all ages, however, it starts to get more common after the age of 50. Although it is more common in smokers around one in eight people with lung cancer never smoked.

Finding lung cancer early improves the chances of successful treatment.

So if you have a cough for three weeks or more, it's worth contacting your GP to be on the safe side.

...about how to spot it

You need to tell a doctor straight away if you have been coughing for the past three weeks or more. Some of the other symptoms of lung cancer include:

- A cough that has got worse or changes
- Repeated chest infections
- Coughing up blood
- Breathlessness
- Feeling more tired than usual for some time
- Losing weight for no obvious reason
- An ache or pain in your chest or shoulder that has lasted some time.

If you notice any of these symptoms, contact your doctor right away.

...about how important it is to tell your doctor

Detecting lung cancer early makes it easier to treat, so telling your doctor quickly may save your life. It's probably nothing serious but it could also be a sign of something else that needs treatment.

So, don't ignore the symptoms or put off telling your doctor. It can make the world of difference and you won't be wasting anyone's time.

And if you know anyone who has any of these symptoms, insist they tell their doctor. Again, it's probably nothing serious, but they should get it checked out.

...about telling your doctor

Your doctor will ask you a few questions, like the ones below, and may suggest a chest x-ray. This is standard procedure and nothing to worry about. Taking an x-ray is quick and simple and doesn't require an overnight hospital stay.

Your doctor might ask you some of these questions:

- How long have you had a cough?
- Has your cough changed over time?
- Have you coughed up any blood?
- Have you had any chest infections recently?
- Have you been short of breath? For how long?
- Has your shortness of breath changed over time?
- Have you lost any weight?
- Have you had a pain in your chest or shoulder?
- Has this pain changed over time?

It may help to write down your symptoms and how you feel, so you don't forget anything when telling your doctor.

...about reducing your risk of cancer

You can reduce the risk with lifestyles changes, including;

Stop smoking
Smoking increases the risk of many cancers. If you smoke, the best thing you can do for your health is to quit. There's plenty of support and help from the NHS. Visit **stopsmokingwales.com** or call freephone **0800 085 2219**.

Look after yourself
Being overweight or obese can increase your risk of some cancers. Try to maintain a healthy weight and keep active. Swimming, cycling, dancing, walking – the more you do, the better. Try to eat a healthy, balanced diet too, with plenty of fruit and vegetables.

Cut down on alcohol
Drinking too much alcohol can lead to a number of health problems and is linked with some cancers. By drinking less, you'll reduce your health risks.

...about how telling your doctor early could save your life

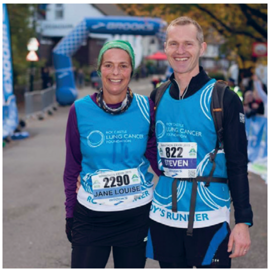
Jane Holmes

"I was diagnosed with lung cancer in June 2014. I was living a healthy lifestyle, running regularly, eating well and didn't smoke. As you can imagine, the diagnosis and prognosis came as a shock.

I have since had chemotherapy, radiotherapy and then became eligible for surgery. I had surgery in June 2015 and four months later completed the Snowdonia marathon! I have had clear scans since surgery and have been on several lovely holidays and have entered for the marathon again this year!

I am being monitored regularly but am delighted to be doing so well."

"So here I was, seven years down the line, after stopping smoking following 50 years of the habit. I was bullet proof, feeling good and looking forward to the rest of my life following retirement. In February 2014, with no warning, I suffered a bout of pneumonia and following treatment to clear that I was diagnosed with lung cancer. Two years after treatment I now feel good, and so people tell me, look well. Never think 'it won't happen to me', never think 'I am afraid to seek help'. There are wonderful people who give amazing treatments and care out there. You won't be alone, you go through it together."

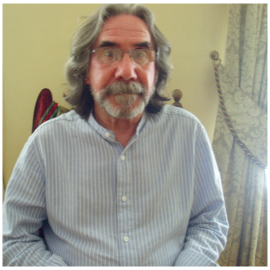
Graham Thomas

Unclear on anything?

Visit **go.nhs.wales/lungcancer**

CANCER RESEARCH UK

Ffonio'n unig sydd angen
0808 808 1010
Ffoniwch ni, 365 diwrnod y flwyddyn

tenovus cancer care gofal canser

Community Pharmacy Wales Fferylliaeth Gymunedol Cymru

Cefnogir gan Lywodraeth Cymru Supported by Welsh Government

GIG CYMRU NHS WALES

BE CLEAR ON CANCER

GIG CYMRU
NHS WALES

You need to tell your doctor straight away if you have any of the following symptoms:

- **A cough that has lasted for 3 weeks or more**
- **Coughing up blood**
- **Breathlessness**
- **Repeated chest infections**

go.nhs.wales/lungcancer

BOD YN GLIR AM GANSER

GIG CYMRU NHS WALES

Mae’n rhaid i chi ddweud wrth eich meddyg ar unwaith os oes gennych chi unrhyw rai o’r symptomau canlynol:

- **Peswch sydd wedi para am 3 wythnos neu fwy**
- **Pesychu gwaed**
- **Colli eich gwynt**
- **Haint dro ar ôl tro ar y frest**

ewch.gig.cymru/canseryrysgyfaint

Supplementary File 6. First treatment definitive received for lung cancer

	2015 Number of cases (%)	2016 Number of cases (%)	% Change	p-value
**Active monitoring**				
Pre-campaign	55 (14.7%)	64 (17.2%)	2.5%	
Campaign	66 (16.5%)	70 (17.0%)	0.5%	0.84
Post-campaign	59 (15.4%)	56 (15.7%)	0.3%	
**Chemotherapy**				
Pre-campaign	88 (22.9%)	100 (26.8%)	3.9%	
Campaign	107 (26.7%)	89 (21.6%)	-5.1%	0.09
Post-campaign	85 (22.1%)	67 (18.8%)	-3.4%	
**Radiotherapy**				
Pre-campaign	70 (18.7%)	69 (18.5%)	-0.2%	
Campaign	71 (17.7%)	81 (19.7%)	2.0%	0.48
Post-campaign	70 (18.2%)	60 (16.8%)	-1.4%	
**Specialist Palliative Care**				
Pre-campaign	67 (17.9%)	49 (13.1%)	-4.7%	
Campaign	72 (18.0%)	55 (13.3%)	-4.6%	0.07
Post-campaign	55 (14.3%)	63 (17.6%)	3.3%	
**Surgical resection**				
Pre-campaign	60 (16.0%)	52 (13.9%)	-2.1%	
Campaign	57 (14.2%)	72 (17.5%)	3.3%	0.20
Post-campaign	79 (20.6%)	72 (20.2%)	-0.4%	
**Other**				
Pre-campaign	11 (2.9%)	18 (4.8%)	1.9%	
Campaign	10 (2.5%)	25 (6.1%)	3.6%	0.01
Post-campaign	17 (4.4%)	14 (3.9%)	-0.5%	
**Any treatment**				
Pre-campaign	349 (93.1%)	352 (94.4%)	1.3%	
Campaign	383 (95.5%)	392 (95.1%)	-0.4%	0.81
Post-campaign	365 (95.1%)	332 (93.0%)	-2.1%	
**No treatment**				
Pre-campaign	26 (6.9%)	21 (5.6%)	-1.3%	
Campaign	18 (4.5%)	20 (4.9%)	0.4%	0.81
Post-campaign	19 (4.9%)	25 (7.0%)	2.1%	

Supplementary File 7. Performance status of lung cancer patients

	2015<br>Number of cases (%*)	2016<br>Number of cases (%*)	% Change	p-value
**Performance status group 0 and 1**				
Pre-campaign	176 (47.2%)	156 (43.5%)	-3.7%	
Campaign	199 (49.8%)	181 (46.6%)	-3.1%	0.38
Post-campaign	195 (51.3%)	159 (46.6%)	-4.7%	
**Performance status group 2**				
Pre-campaign	87 (23.3%)	99 (27.6%)	4.3%	
Campaign	90 (22.5%)	100 (25.8%)	3.3%	0.28
Post-campaign	74 (19.5%)	77 (22.6%)	3.1%	
**Performance status group 3 and 4**				
Pre-campaign	109 (29.2%)	103 (28.7%)	-0.5%	
Campaign	109 (27.3%)	107 (27.6%)	0.3%	0.92
Post-campaign	108 (28.4%)	105 (30.8%)	2.4%	
**Unknown**				
Pre-campaign	2	14		
Campaign	1	24		
Post-campaign	4	16		

*Percentage of known cases
